# Does a Classroom Standing Desk Intervention Modify Standing and Sitting Behaviour and Musculoskeletal Symptoms during School Time and Physical Activity during Waking Time?

**DOI:** 10.3390/ijerph15081668

**Published:** 2018-08-06

**Authors:** Jolyn Ee, Sharon Parry, Beatriz IR de Oliveira, Joanne A. McVeigh, Erin Howie, Leon Straker

**Affiliations:** Curtin University, Kent Street, Bentley, Perth 6102, Western Australia, Australia; s.ee3@graduate.curtin.edu.au (J.E.); Beatriz.Oliveira@curtin.edu.au (B.I.d.O.); Joanne.McVeigh@curtin.edu.au (J.A.M.); erin.howie@curtin.edu.au (E.H.); L.Straker@curtin.edu.au (L.S.)

**Keywords:** sedentary behaviour, standing desks, physical activity, musculoskeletal discomfort, children

## Abstract

Children are increasingly spending more time sedentary at school and during leisure time. This study examined the effects of a standing desk intervention in a classroom on children’s standing and sitting time at school, sedentary and physical activity levels throughout the day (waking hours), and musculoskeletal discomfort. A within-subjects crossover study design was used. Participants used either a standing desk or traditional seated desk for 21 days before swapping desks for another 21 days. Accelerometry and musculoskeletal discomfort data were collected during the last seven days of each 21-day period. Mixed models were used to analyse accelerometry data. Zero-inflated regression models and logistic regression models were used to analyse discomfort data. Forty-seven male students (aged 10–11 years) participated in the study. Standing time was 21 min/school day higher (*p* < 0.001) and sitting time was 24 min/school day lower (*p* = 0.003) when standing desks were used. No significant differences were found in sedentary and physical activity time during waking hours between the standing desk and seated desk conditions. Students were less likely to report musculoskeletal discomfort in the neck, shoulder, elbows and lower back when using standing desks (OR 0.52–0.74). Standing desks significantly increased classroom standing time and decreased musculoskeletal discomfort reports but had no overall effect on daily physical activity levels. Schools should consider moving towards classrooms enabling a variety of postures to potentially improve the long-term health of children.

## 1. Introduction

The Australian Physical Activity and Sedentary Behaviour Guidelines for children aged 5 to 12 years (2017) recommend that children should move more and sit less, and to limit their screen time to a maximum of two hours daily [1]. However, a large proportion of children fail to meet this recommendation [2,3]. Many still spend a large proportion of the day being sedentary [4,5]. Modifying sedentary behaviours in children is important as activity and behavioural patterns formed during childhood can follow through to adolescence and adulthood [6,7].

High levels of sedentary time may lead to negative health effects in children. Sedentary behaviour, defined as “any waking behaviour characterised by an energy expenditure ≤ 1.5 metabolic equivalents, while in a sitting, reclining or lying posture” [8], may increase the risk of cardiometabolic disease, such as hypertension and diabetes, in children [5,9,10]. The risk of childhood obesity increases in a dose-response manner as sedentary levels rise [5,11,12]. Excessive screen time (often used as a proxy for sedentary time in children) is also associated with depressive symptoms whereas reduced screen time and increased physical activity levels are linked to emotional stability, higher cognitive function and higher self-esteem in children [5,12,13].

Greater sedentary time is associated with a higher prevalence of musculoskeletal pain/discomfort amongst children and adolescents [14,15,16]. The excessive use of tablets and computers can lead to increases in sedentary time and undesirable postures from a young age [15,16,17]. Consequently, a higher prevalence of musculoskeletal discomfort, such as neck or back pain, have been found in children [14,15,17]. Musculoskeletal discomfort developed during childhood may progress to become chronic musculoskeletal pain syndromes in adulthood [18].

Schools have great potential to positively influence children’s physical activity and health behaviours [19,20]. There have been a number of school-based interventions aimed at increasing physical activity in students such as integrating physical activity into the delivery of academic material [21,22] and the implementation of activity breaks [23]. These approaches have been effective at improving physical activity in children [24]. However, children spend many hours sitting at desks during school hours [19,20]. Due to time constraints, budget cuts and a growing focus on standardised teaching and academic achievement, physical activity levels in schools have declined over recent years [25,26,27]. Increasing sedentary time and decreasing physical activity may hinder the very academic success that schools strive to attain [28,29]. As such, it is important not to compromise on time spent on the academic curriculum when implementing physical activity interventions in schools [27].

Replacing traditional seated desks and chairs in classrooms with standing desks could potentially reduce sedentary time and increase standing time and light activity. In a recent review, it was found that standing desks in classrooms can increase standing time by 24–40 min per school day and decrease sitting time by 59–64 min per day [30]. However, only a few studies have explored the effects of standing desks on musculoskeletal discomfort in children and results from these studies were inconsistent [31,32]. Given that research that has examined the association of occupational standing with musculoskeletal symptoms in adults has found that prolonged standing in adults is associated with the development of low back pain [33], it is important to examine the relationship between standing and musculoskeletal symptoms in children. If standing desks prove to be effective in increasing standing time and decreasing sitting time in children, while not causing excessive musculoskeletal discomfort, schools would have more reasons to implement these desks in classrooms. This could lead to significant positive changes in the long-term health of children.

In addition, most of the studies investigating the effects of standing desks in classrooms have only measured sedentary time and physical activity outcomes during school hours, rather than over the whole day. It is important to analyse children’s sedentary time and physical activity throughout the whole day, as school only takes up a portion of the day, and changing children’s behaviour in school may have consequences in non-school hours.

The purpose of this study was to determine the short-term effects of a standing desk intervention in a classroom on students’ (a) standing and sitting time during school hours; (b) physical activity and sedentary time during all waking hours; and (c) musculoskeletal discomfort.

## 2. Materials and Methods

### 2.1. Study Design

This study was a within-subjects crossover trial, whereby each participant acted as his own control. The school funded the purchase of standing desks (AlphaBetter^®^ Adjustable-Height Stand-Up Desk, SAFCO Products Australia^®^ at a cost of $750 AUS/desk) for half a classroom. The standing desks used in this study had adjustable heights and “fidget bars”. Fidget bars allowed students to swing their non-supporting leg while standing. In both 2016 and 2017, the class was divided into two groups—one group used the standing desks while the other group used the traditional seated desks. After 21 school days (three rotations of the school’s seven-day timetable), the students changed desks. Students who had used the traditional seated desks previously started using the standing desks and vice versa. Students remained in this condition for a further 21 school days. Hip and thigh accelerometer data, and musculoskeletal discomfort ratings were collected on the last seven days of each 21-school-day period.

The study was approved by the Human Research Ethics Committee at Curtin University (Approval number: RDHS-157-16).

### 2.2. Participants

Participants were a convenience sample of male students from two Grade 4 classes at an all-boys private school in Perth, Western Australia. Data from each class were collected at separate time periods during 2016 and 2017. All children in the classes were invited to participate. Exclusion criteria were physical health problems that precluded standing for extended periods of time, or inability to wear accelerometers around their hips and thighs.

### 2.3. Outcome Measures

#### 2.3.1. Accelerometer Data

ActiGraph GT9X Link accelerometers (Actigraph LLC, Pensacola, FL, USA) were used to measure participants’ standing and sitting time during school hours, and sedentary time and physical activity levels during waking hours. Each participant wore two accelerometers—one on the thigh and one on the hip. Data were collected at 30 Hz, and then converted to counts per 15 s epochs for analysis. Short epochs allow for the recording of brief sporadic bursts of high-intensity activities, which are commonly observed in children [34].

The thigh-worn accelerometer was used to estimate time in standing and sitting during the participants’ school hours. When mounted on the thigh, the Actigraph inclinometer function has been found to be a valid and reliable method of assessing standing and sitting postures [35,36]. The thigh-worn accelerometers were attached upon arrival at school and removed just prior to the participants leaving school at the end of the school day. Non-wear time was detected by visual examination of the inclinometer data.

Hip-worn accelerometers measured participants’ physical activity and sedentary time, during all waking hours. To date, the validity of the ActiGraph GT9X Link accelerometers to determine physical activity intensities has not been determined in children. However, ActiGraph GT3X+ accelerometers (ActiGraph LLC, Pensacola, FL, USA), which use the same software and hardware as the ActiGraph GT9X Link accelerometers, have been shown to be valid devices in assessing physical activity and sedentary behaviour intensity in children and youth [37,38]. The hip-worn accelerometers were held in place by a belt for seven days (24 h/day), except during showers/baths and water-based activities like swimming.

Waking wear time from the hip-worn accelerometers was determined by visual inspection of the ActiGraph files by a trained rater and a customised algorithm (SAS Version 9.3, SAS Institute, Cary, NC, USA) [39]. Waking wear data were processed using ActiLife version 6 (ActiLife software; Pensacola, FL, USA) using Evenson’s accelerometer cut-points for children [40] to estimate the amount of time spent during waking hours in different levels of physical activity intensity (light, moderate and vigorous) and sedentary time. Accelerometry data for a participant was included in the study if there was a minimum wear time of ten hours/day for at least one day [41].

#### 2.3.2. Musculoskeletal Discomfort

Participants completed a modified version of the Nordic Musculoskeletal Questionnaire [42] twice daily. One assessment was completed in the early part of the school day and the other in the later part of the school day. This assessment of musculoskeletal discomfort has been used extensively in studies involving children [32,43]. The Nordic Musculoskeletal Questionnaire comprises a body map with nine labelled body parts and a numeric intensity rating scale from zero to ten [44]. Participants rated discomfort levels felt at the following body parts—neck, shoulders, elbows, wrists/hands, upper back, lower back, hips/thighs, knees and ankles/feet [42].

### 2.4. Analysis

Linear mixed models were used to examine the differences in the average standing and sitting times (at school) and the average times spent sedentary and in various physical activity intensities per day (waking hours) between the standing desk and seated desk conditions. These models included all valid observed data and were adjusted for the number of minutes the accelerometers were worn.

Musculoskeletal discomfort ratings at each body part were zero-skewed and, after transforming to a zero to one scale, compared between the standing desk and seated desk conditions using zero-inflated regression models, clustering on participant to account for inter-dependence between individual participant’s repeated measures. Model results were summarised using regression coefficients, predicted marginal means and their 95% confidence intervals (CI). Because discomfort frequencies greater than 0 were very low, discomfort ratings were also recoded to “presence of discomfort” (>0) and “absence of discomfort” (= 0). The effect of using a standing desk compared to a seated desk on the likelihood of discomfort was evaluated using logistic regression models with model results summarised using odds ratios (OR) and their 95% CI. All p-values were two-sided, and *p*-values < 0.05 were considered statistically significant. Stata/IC v15.0 (StataCorp, College Station, TX, USA) was used to analyse the data.

## 3. Results

A convenience sample of 48 Grade 4 male students, aged ten to 11 years were asked to participate in this study over 2016–17. One student declined to participate so a total of 47 students participated in the study. None of the participants reported injuries or physical health conditions at the time of enrolment.

### 3.1. School Standing and Sitting Time

**Figure 1:**
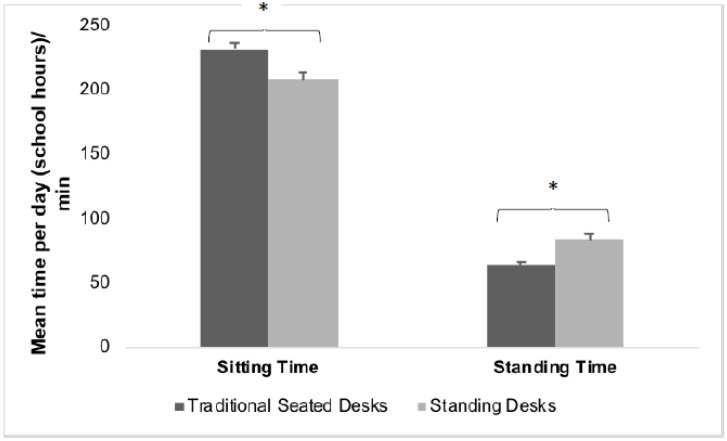
Comparison of mean (standard deviation (SD)) sitting and standing time between standing desk and seated desk conditions during school time. Note: * = *p* < 0.05 (significant difference between conditions).

Wear time for the thigh-worn accelerometers was six hours/day while at school. During school hours, more time was spent sitting (61%) than standing (19%) regardless of whether standing desks or traditional seated desks were used (Figure 1). However, mean (standard error (SE)) standing time at school was 84 (4) min/day (school time) in the standing desk condition compared to 63 (3) min/day (school time) in the seated desk condition (mean difference (SE) = 20 (4) min/day (school time), *p* < 0.001). Mean (SE) sitting time was 208 (6) min/day (school time) in the standing desk condition and 231 (5) min/day (school time) in the seated desk condition (mean difference (SE) = 24 (8) min/day (school time), *p* = 0.003).

### 3.2. Whole Day Physical Activity and Sedentary Time

Children spent approximately 68% of their day sedentary behaviour, 26% in light activity and around 6% of the waking day being moderately or vigorously active. There were no significant differences in the time the participants spent engaged in sedentary behaviour, light activity, moderate activity or vigorous activity during waking time, regardless of desk type used (*p* > 0.05) (Table 1).

### 3.3. Musculoskeletal Discomfort

More than 80% the participants (85% standing and 81% sitting) reported discomfort in one or more body part during school time on one or more days. As seen in Table 2, there was a small but statistically significant reduction in mean neck discomfort in the standing desk condition compared to the seated desk condition (β = −0.280; *p* = 0.005). Mean neck discomfort ratings indicate a mean difference of 0.21/10 less in the standing desk condition. There were no differences in discomfort levels at the other body parts.

**Figure 2:**
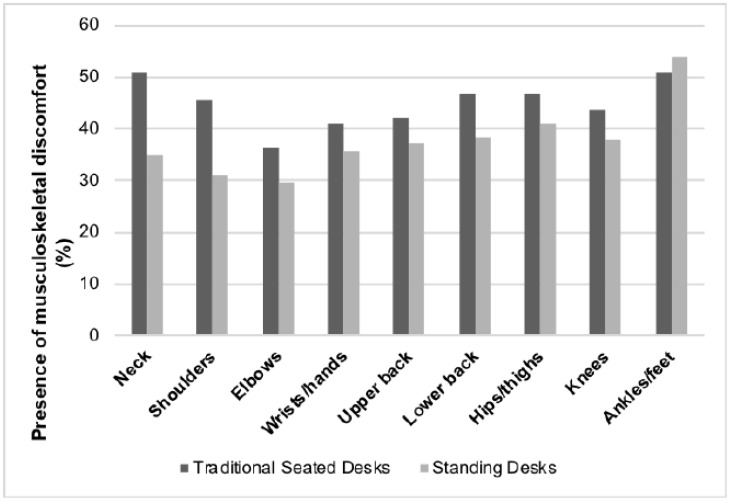
Percentage of participants reporting the presence of musculoskeletal discomfort at various body parts between the standing desk and seated desk conditions.

Figure 2 illustrates the percentage of participants reporting the presence of musculoskeletal discomfort at each body part. With the exception of the ankles/feet, the proportion of participants reporting discomfort in the various body parts was less in the standing desk condition compared to the seated desk condition.

Logistic regression on discomfort presence (Table 2) indicated that the use of standing desks when compared to traditional seated desks reduced the likelihood of participants reporting musculoskeletal discomfort at the neck, shoulders, elbows and lower back. The OR for ankles/feet discomfort is >1 but was not statistically significant (*p* = 0.350).

## 4. Discussion

The present study found that the use of standing desks increased standing time and reduced sitting time at school. There were no concurrent changes in the time spent sedentary or in light or moderate/vigorous physical activity intensity levels during waking hours. There was also a lower rating of neck discomfort intensity and an overall lower likelihood of reporting neck, shoulders, elbows and lower back discomfort when using the standing desk.

The use of standing desks in classrooms had a significant impact on standing and sitting time. In this study, participants in the standing desk condition stood about 20 min more and sat about 24 min less at school, compared to during the seated desk condition. This is consistent with findings from previous studies [30,31,32,43,45]. Aminian, Hinckson and Stewart [32] reported an increase in standing time by 24 min/day (school hours) after 9 weeks of using standing desks, compared to baseline. In that study the school hours (six hours) and participant age group were the same as the present study [32]. Significant decreases in sitting time with the use of standing desks were also found in previous studies, whether the study investigated sitting time during school hours or sitting time during waking hours [43,45]. Clemes et al. found that sitting time decreased by 52 min/school day in the UK and 44 min/school day in Australia, when comparing pre- and post-intervention [45]. Hinckson et al. reported that children who had used standing desks at school sat (497 min/day) less than those who did not use standing desks (540 min/day) [43].

The current study was different from previous studies as they primarily investigated changes in standing and sitting time pre- and post-intervention whereas the present study compared standing and sitting time between the standing desk and seated desk conditions post-intervention. Despite these differences, both past and present studies show statistically significant increases in standing time and decreases in sitting time with the use of standing desks in classrooms, supporting that standing desks may help increase standing time and decrease sitting time in children.

Although the use of standing desks had a significant effect on standing and sitting time during school hours, there was no statistically significant or clinically meaningful difference in the sedentary time and light and moderate/vigorous physical activity level time between the standing desk and seated desk conditions during all waking hours. A recent review that explored the impact of standing desks on sedentary time and physical activity time in children found inconsistent results for school time effects [30]. Some studies found that stepping time and total step count did not increase with the use of standing desks when compared to baseline measurements or the control group [32,43,45,46]. These findings are consistent with the findings of the present study.

It may be that when participants were standing longer at school, there was a compensation of being more sedentary after school. For example, some studies have found that an increase in physical activity at one part of the day tends to be followed by a reduction in physical activity at another part of the day in children [47,48]. Conversely, some studies have shown no compensatory behaviour with children staying active after school, even if they were more active at school [31,32,49]. Further investigation is needed to determine if there is a consistent compensatory behaviour over time.

This study also found that the proportion of sedentary and light activity times was similar in the standing desk and seated desk conditions. It could be that the hip-worn accelerometers may have detected standing still and weight shifting as sedentary behaviour, potentially overestimating sedentary levels and underestimating light activity levels in the standing desk condition. This could have resulted in this study finding no differences in sedentary and light activity levels between both conditions.

In addition, the use of the fidget bars (leg swinging) could have been categorised as sedentary behaviour rather than light activity too, since movement would have occurred primarily at or below the knee, rather than at the hip. Once again, this could have led to an overestimation of sedentary levels when using the standing desk. Future studies can consider placing the accelerometers at a different body part to detect the use of the fidget bars as light activity.

Previous studies investigating the impact of standing desks on musculoskeletal discomfort in children have found inconsistent results, with some reporting no significant differences in musculoskeletal discomfort [32] and some reporting more neck and back discomfort with the use of standing desks [31]. The present study found a lower intensity of neck discomfort and a lower likelihood of participants reporting neck, shoulders, elbows and lower back discomfort in the standing desk condition. It may be that participants had their necks/trunks in mid-range flexion when using the standing desks, rather than extreme neck/trunk postures when sitting, which may be associated with neck pain [50,51] and contribute to the reduced intensity of neck discomfort in the standing desk condition. Although neck discomfort ratings were significantly lower in the standing desk condition, the actual mean difference was 0.21/10, which may not be clinically meaningful, especially in acute conditions.

The lower likelihood of participants reporting lower back discomfort in the standing desk condition could potentially be explained by the association of sedentary behaviour, such as sitting and engaging in screen-based activities, with lower back pain in adolescents [14,16]. Studies investigating the impact of sedentary behaviour on lower back discomfort in children specifically have not found clear results [31,32]. Although this study did not find a difference in sedentary time during waking hours between the standing desk and seated desk conditions, a decrease in classroom sitting time was found in the standing desk condition. This study seems to suggest that the association between lower back discomfort and sitting may also be present in this population.

An interesting finding of this study was that there were no significant differences in musculoskeletal discomfort ratings and likelihood of participants reporting musculoskeletal discomfort in the lower limb joints between the standing desk and seated desk conditions. This suggests that spending more time standing at school may not cause additional lower limb discomfort, even though these joints take more load in standing and prolonged occupational standing is associated with lower limb and back discomfort [33]. Weight shifting or the use of the fidget bar could have contributed to this. Alternatively, the participants in this study may not have used the standing desks for prolonged periods of time because some lessons were not conducted in the main classroom where the standing desks were located. Thus, students may have had greater postural variety when in the standing desk condition, which is suggested to reduce musculoskeletal symptoms [52].

Overall, the current study shows that standing desks do not exacerbate musculoskeletal discomfort in children and may even help alleviate discomfort at some body parts. Given that musculoskeletal discomfort does not increase and children may have more varied postures when standing desks are used, the likelihood of children developing musculoskeletal pain may decrease, thereby also decreasing the risk of developing chronic musculoskeletal discomfort in adulthood [18].

Despite these positive findings regarding the use of standing desks in schools, the financial cost of providing standing desks may not be achievable in many schools. Future research is also needed to explore the long-term effects of using standing desks in classrooms on children, and this may then help weigh up the long-term costs and benefits of using standing desks in schools, giving schools a clearer idea of whether it is worth moving towards standing classrooms.

A limitation of the present study is the low generalisability to other school-aged children. The participants were all boys, aged 10 to 11 years, who were enrolled in a private school. Physical activity opportunities may be different between private and public schools, and between male and female students. Also, the validity of the accelerometers used has not been determined in the age group of our participants. However, these accelerometers have the same software and hardware as other accelerometers that already have their validity shown in children [37,38,41].

A strength of the present study was the within-subjects crossover study design, which reduced the influence of confounding covariates, since each participant acted as his own control. The current study also had two separate cohorts of students who underwent a replicated study procedure, adding power and confidence to our findings. Lastly, thigh-worn accelerometers were used to measure standing and sitting time, which is best practice [36].

## 5. Conclusions

The use of standing desks in classrooms contributed to an increase in classroom standing time and a decrease in classroom sitting time, as well as reducing the likelihood of neck, shoulders, elbows and lower back discomfort in children. Standing desks may not affect children’s overall sedentary and physical activity time adversely. The installation of standing desks in classrooms has potential health benefits for children and may encourage the habit of sitting less which could potentially be carried into adulthood.

## Figures and Tables

**Table 1:** Comparison of mean (SE) sedentary time and time spent in light, moderate and vigorous physical activity during waking time between standing desk and seated desk conditions.

	Mean (SE) (Waking Time)	Standing Desks	Seated Desks	*p*-Value
Mean (SD) number of wear days		4 (2)	4 (2)	
Mean (SD) total waking time/day (min)		972 (160)	1004 (167)	
Sedentary	Min/day	674 (23)	686 (26)	0.790
	Percentage/day	68.7 (1.1)	67.3 (1.3)	
Light Activity	Min/day	241 (7)	256 (6)	0.111
	Percentage/day	25.3 (0.8)	26.2 (0.9)	
Moderate Activity	Min/day	39 (2)	42 (2)	0.260
	Percentage/day	4.1 (0.2)	4.4 (0.3)	
Vigorous Activity	Min/day	18 (1)	20 (1)	0.330
	Percentage/day	2.1 (0.3)	2.0 (0.2)	

**Table 2:** Comparison of B coefficients, predicted mean and odds ratios between standing desk and seated desk conditions.

Body Part	Condition	Transformed Pain Scores Using a Zero One Inflated Beta Distribution					Logistic Regression on Pain Indicator		
		B Coefficient (Pain Score)	SE(B)	Predicted Mean	95% CI Mean	*p*-Value	OR (Outcome = Any Pain)	95%CI OR	*p*-Value
Neck	Sit			1.31	0.81–1.81		1.00		
	Stand	−0.280	0.100	1.10	0.67–1.60	0.005	0.52	0.41–0.67	<0.001
Shoulders	Sit			1.23	0.68–1.79		1.00		
	Stand	−0.150	0.102	1.13	0.67–1.60	0.142	0.54	0.42–0.70	<0.001
Elbows	Sit			1.02	0.55–1.49		1.00		
	Stand	−0.092	0.106	0.97	0.50–1.45	0.384	0.74	0.57–0.96	0.023
Wrists/hands	Sit			1.25	0.69–1.82		1.00		
	Stand	−0.084	0.104	1.20	0.68–1.71	0.419	0.80	0.62–1.03	0.089
Upper back	Sit			1.30	0.83–1.77		1.00		
	Stand	−0.132	0.100	1.21	0.74–1.68	0.189	0.81	0.63–1.04	0.103
Lower back	Sit			1.42	0.85–1.98		1.00		
	Stand	−0.111	0.103	1.33	0.80–1.86	0.281	0.71	0.55–0.91	0.007
Hips/thighs	Sit			1.53	0.98–2.08		1.00		
	Stand	−0.177	0.097	1.38	0.90–1.87	0.07	0.79	0.62–1.02	0.070
Knees	Sit			1.40	0.79–2.02		1.00		
	Stand	−0.172	0.121	1.28	0.77–1.79	0.156	0.80	0.62–1.03	0.079
Ankles/feet	Sit			2.07	1.38–2.76		1.00		
	Stand	−0.179	0.123	1.90	1.28–2.52	0.145	1.12	0.88–1.44	0.350
